# Cross-modal plasticity in children with cochlear implant: converging evidence from EEG and functional near-infrared spectroscopy

**DOI:** 10.1093/braincomms/fcae175

**Published:** 2024-05-21

**Authors:** Mickael L D Deroche, Jace Wolfe, Sara Neumann, Jacy Manning, Lindsay Hanna, Will Towler, Caleb Wilson, Alexander G Bien, Sharon Miller, Erin Schafer, Jessica Gemignani, Razieh Alemi, Muthuraman Muthuraman, Nabin Koirala, Vincent L Gracco

**Affiliations:** Department of Psychology, Concordia University, Montreal, Quebec, Canada, H4B 1R6; Hearts for Hearing Foundation, Oklahoma City, OK 73120, USA; Hearts for Hearing Foundation, Oklahoma City, OK 73120, USA; Hearts for Hearing Foundation, Oklahoma City, OK 73120, USA; Hearts for Hearing Foundation, Oklahoma City, OK 73120, USA; Hearts for Hearing Foundation, Oklahoma City, OK 73120, USA; Department of Otolaryngology, University of Oklahoma Health Sciences Center, Oklahoma City, OK 73104, USA; Department of Otolaryngology, University of Oklahoma Health Sciences Center, Oklahoma City, OK 73104, USA; Department of Audiology & Speech-Language Pathology, University of North Texas, Denton, TX 76201, USA; Department of Audiology & Speech-Language Pathology, University of North Texas, Denton, TX 76201, USA; Department of Developmental and Social Psychology, University of Padova, 35131 Padua, Italy; Department of Psychology, Concordia University, Montreal, Quebec, Canada, H4B 1R6; Section of Neural Engineering with Signal Analytics and Artificial Intelligence, Department of Neurology, University Hospital Würzburg, 97080 Würzburg, Germany; Haskins Laboratories, New Haven, CT 06511, USA; Haskins Laboratories, New Haven, CT 06511, USA

**Keywords:** cochlear implant, neuroplasticity, brain imaging, language development, children

## Abstract

Over the first years of life, the brain undergoes substantial organization in response to environmental stimulation. In a silent world, it may promote vision by (i) recruiting resources from the auditory cortex and (ii) making the visual cortex more efficient. It is unclear when such changes occur and how adaptive they are, questions that children with cochlear implants can help address. Here, we examined 7–18 years old children: 50 had cochlear implants, with delayed or age-appropriate language abilities, and 25 had typical hearing and language. High-density electroencephalography and functional near-infrared spectroscopy were used to evaluate cortical responses to a low-level visual task. Evidence for a ‘weaker visual cortex response’ and ‘less synchronized or less inhibitory activity of auditory association areas’ in the implanted children with language delays suggests that cross-modal reorganization can be maladaptive and does not necessarily strengthen the dominant visual sense.

## Teaser

Deafness early in life should promote vision, but this cross-modal plasticity does not necessarily support language. Using electroencephalography and functional near-infrared spectroscopy, Deroche *et al*. report weaker visual cortex activity elicited by a chequerboard (and less coupled with auditory cortex) for children with cochlear implants who exhibit poor language outcomes.

## Graphical Abstract

**Graphical Abstract:**
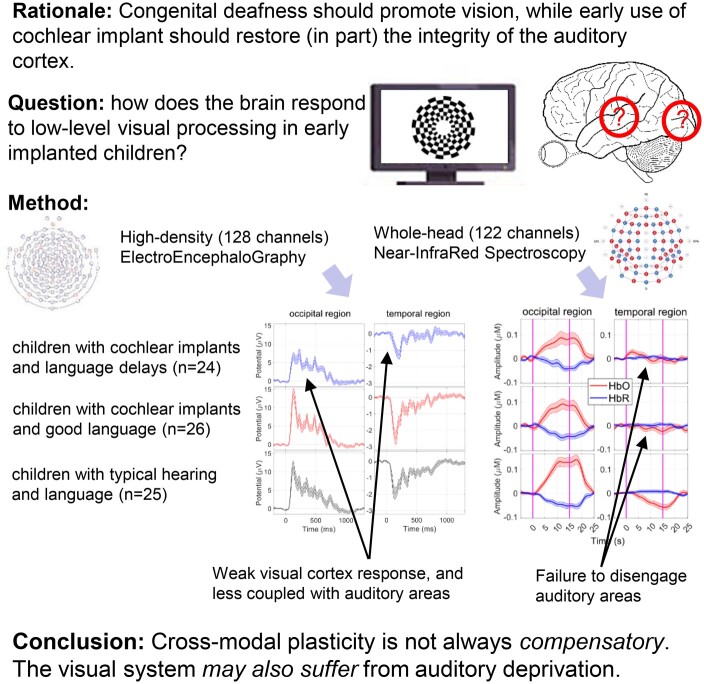

## Introduction

In response to sensory deprivation, the brain undergoes reorganization to enhance another sense, typically vision, hearing or somatosensory processing, which has been extensively studied. The extent of this reorganization increases in proportion to the onset and duration of the deprivation.^1^ For example, following congenital blindness, the occipital cortex may be recruited for Braille reading, to discriminate vibrotactile stimuli,^2,3^ localize sounds,^4^ process spoken language^5^ and support verbal memory.^6^ Following congenital deafness, the auditory cortex may be recruited for sign language,^7^ specific visual tasks or movements^8-10^ and the processing of vibrotactile stimuli.^11^ These instances of ‘cross-modal plasticity’ highlight the remarkable adaptability of the brain to various environments, especially during early development.

Although not always explicitly stated, the term ‘adaptation’ is often presented in a positive light: a key feature of the cross-modal changes aforementioned is that the brain ‘compensates’ for the deprivation of one sense with ‘enhanced abilities’ in another. But this view somewhat conflicts with our current understanding of brain development where interconnected networks develop and specialize in tandem. This has been formulated by the interactive specialization framework.^12^ Especially for complex functions such as language processing, which is inherently multimodal and integrative,^13^ the auditory and visual systems need to support each other rather than compete. From this perspective, one might expect the brain to ‘suffer’ from the deprivation of one sense with ‘poorer abilities’ in another. To draw a simple analogy, think of a table with a missing leg: aside from being fragile, it might also put extra strain on the remaining legs making them weaker, not stronger.

One key in understanding why networks specialize in a certain way is discerning the periods during which they are notably susceptible to experiential influences, i.e. sensitive periods.^14,15^ For example, around 1 year of age, there is a transition from children predominantly focusing on the eyes of a speaker to directing their attention towards the speaker’s mouth,^16^ which could be indicative of children starting to attend to the way speech is produced. Around the same time, a window for discrimination of native versus non-native speech sounds closes.^17^ From the sensorimotor coupling model of speech development,^18^ we could imagine how visual articulatory cues would consolidate production behaviours that seem native versus foreign (or abnormal). In other words, both phenomena could be related to the foundations of future lip-reading skills. If that is the case, then auditory deprivation might not necessarily facilitate lip-reading (because the ability to recognize speech visually often necessitates solid predictions about the speech sounds resulting from a given articulatory behaviour). This is where children with cochlear implants (CIs) happen to be a population of choice in this scientific endeavour because they allow for the environment to change suddenly (e.g. the world no longer being silent) as a particular window of plasticity closes.

Today, in many cases of congenital deafness, children may receive a CI as early as 1 year of age (occasionally even earlier), and their hearing recovers impressively. But among children with CI in general (not only those implanted at 1 year of age), there is a large variability in outcomes that remains unexplained. Typically, multi-factorial models account for 50% of the variance,^19,20^ and substantially less in adults,^21,22^ using a combination of personal characteristics (cognitive skills, non-verbal intelligence and inherent language aptitudes), device parameters (electrode array, quality of mapping and electric dynamic range) and communication mode.^20,23-26^ We strongly suspect that the status of the auditory nerve and auditory brain^27,28^ would be additional factors to further explain why a given child derives much benefit from their device, while another is not, despite both being implanted at a young age.^29,30^

Some aspects of brain reorganization have been explored in CI users. A series of electroencephalography (EEG) studies demonstrated activity in the auditory cortex of CI adults elicited by a visual task.^31-34^ These cross-modal visual-evoked potentials (VEPs) were viewed as ‘undesirable’ because their size was (often) inversely related to speech recognition skills. Comparatively, fewer studies exist in CI children whose findings support the maladaptive nature of cross-modal changes.^35-39^ On the other hand, cross-modal changes have also been associated with ‘positive outcomes’. Using PET, Giraud *et al*.^40^ found increased visual cortex response to sounds over time after implantation with responses tuned to meaningful sounds (i.e. words more than vowels), and Strelnikov *et al*.^41^ found a desirable activation of the visual cortex (to visual speech) for later auditory recovery (both studies in post-lingually deafened individuals). Similarly, using functional near-infrared spectroscopy (fNIRS), studies have found a positive association between visual speech and post-implantation activation of the bilateral superior temporal gyrus (STG)^42,43^ (both in post-lingually deafened CI adults and in pre-lingually deafened CI children). These cortical changes are clearly ‘adaptive’ when considering the large benefit that lip-reading provides to support communication, including in paediatric users.^44^ To some degree, this apparent dichotomy has been driven by the choice of neuroimaging technique. Weaker responses in an event-related potential (ERP) paradigm have traditionally been interpreted as poorer encoding but might on the contrary be a sign of efficiency if one considers the brain adaptation induced by repeated stimuli. If so, the interpretation may be more in line with a haemodynamic technique.^45^ This is the sort of methodological debate that motivated us to combine EEG and fNIRS here to get a comprehensive picture of cross-modal reorganization in this population.

To summarize, CI users exhibit reorganization in both auditory and visual cortices,^45^ but whether these changes are adaptive or maladaptive remains debated. Beyond methodological discrepancies, we suspect that the answer has to do with the pressure that language exerts on the connectivity between the visual and auditory cortices. Using two groups of CI children (all implanted before 4 years of age), some with age-appropriate language skills and some with delays, as well as normally hearing (NH) controls, we investigated the response of the visual and auditory cortices to a low-level visual task. Cross-modal changes (whether they are compensatory or deleterious) would predict a form of recruitment of auditory areas, i.e. synchronized potentials (EEG) or a haemodynamic response (fNIRS) over temporal regions. But critically, compensatory changes would predict a stronger visual cortex response, beneficial in the long term to CI children’s language outcomes, whereas deleterious changes would predict a weaker visual cortex response, detrimental to CI children’s language outcomes.

## Materials and methods

### Participants

Seventy-five children between the ages of 7–18 participated and were split in three groups. Fifty children had CIs without comorbidity, communicating through spoken language primarily. Outcomes of language were used to make a first group with low language (LL) aptitudes and a second the group with high language (HL) aptitudes. Twenty-five children with normal hearing and typical language development served as a control population (group NH). Language aptitudes were evaluated through the Clinical Evaluation of Language Fundamentals (CELF)—fifth edition^46^—standardized at a score of 100. Children in the LL group had CELF scores below 1 SD (<85), while children in the HL group had CELF scores above average (>100). Demographic data revealed that (i) there was no difference in sex between the groups [chi-square test; *χ*^2^(2, *N* = 75) < 0.1, *P* = 0.987] with 12 females/12 males in group LL, 13 females/13 males in group HL and 13 females/12 males in group NH. All children had cis gender. (ii) The groups differed in chronological age [*F*(2,72) = 4.3, *P* = 0.017]: children in the LL group were the oldest (13.9 ± 2.6 years, range 8.6–17.5), followed by the NH group (12.6 ± 3.1 years, range 7.3–17.6) and the HL group (11.5 ± 2.8 years, range 7.5–16.6). This age difference was not intended, but, if anything, it ought to confer some maturational advantage to the LL compared to the HL group, and it was controlled for in the analyses. (iii) Children in the LL group were fitted with a hearing aid ‘at a later age’ than children in HL group [*t*(48) = 3.9, *P* < 0.001; 17.2 ± 10.0 months versus 7.2 ± 7.9 months]. Note that the use of the hearing aid was of limited benefit as all children proceeded to CI candidacy, but this 10-month difference speaks about a differential time at which families began to seek audiological services. (iv) Children in the LL group were implanted ‘at a later age’ than children in the HL group [*t*(48) = 2.3, *P* = 0.027; 27.7 ± 12.7 months versus 20.3 ± 10.1 months]. This 7-month difference might seem small, but at such young ages, we know that this has repercussions for the development of speech recognition skills,^30,47-50^ and we confirmed these repercussions with audiological outcomes as children in the HL group had better recognition of words or sentences in quiet or in noise.^26^ (v) All 26 children from the HL group were implanted on both sides, and 21 of the 24 children in the LL group were too. Time interval between first and second implants did not differ between the two groups [*t*(45) = 0.2, *P* = 0.814] and was about 16.5 ± 24.4 months. (vi) All implanted children were properly fitted (aided thresholds of 20–30 dB between 250 and 6000 Hz), and the manufacturers of the device did not differ between the two groups [*χ*^2^(2) = 1.2, *P* = 0.555 on the right side; *χ*^2^(2) = 0.9, *P* = 0.643 on the left side] with a large majority of devices manufactured by ‘Cochlear’ (45 ‘Cochlear’, 3 ‘Advanced Bionics’ and 1 ‘Med-El’ on the right; 43 ‘Cochlear’, 3 ‘Advanced Bionics’ and 1 ‘Med-El’ on the left). Neither the model, nor the electrode array, nor the signal coding strategy differed between the two groups; the coding strategy being ACE for 90 of them (out of 96).

### Stimuli

Children watched a single type of stimuli: a circular chequerboard,^51^ made of 24 alternated patterns of black and white areas over 360° and six concentric rings. This chequerboard rotated every 125 ms to its mirror image. For EEG data acquisition, the chequerboard was presented for 500 ms, followed by a 1000-ms grey screen, and repeated 200 times, resulting in a 5-min task. This stimulation was designed to elicit two types of VEP: one in response to a ‘pattern onset’ as the chequerboard appeared from a grey background every 1500 ms and the other in response to a ‘pattern reversal’ as the chequerboard rotated. This choice increased our chance of tapping into different aspects of visual processing, more or less prone to adaptation^52^ and inducing different spread of activation.^53^ For fNIRS data acquisition, the chequerboard was presented for 15 s (still rotating at 8 Hz) followed by a 15-s rest and repeated 10 times. Again, this resulted in a 5-min task. All tasks were generated using ‘PsychoPy’ and included triggers at the onset of each visual event.

### Equipment

All data were acquired at ‘Hearts for Hearing’ (https://heartsforhearing.org/) in Oklahoma City. EEG was recorded using a high-density 128-electrode sensor array net placed on the scalp using ‘Electrical Geodesics, Inc.’ (EGI) system (MagstimEGI, Oregon, USA). The impedance was kept under 10 kΩ throughout the recording. The reference electrode was located at Cz. The raw data were sampled at 1000 Hz (‘EGI’ net amps 300 system) and stored for offline analysis. Continuous fNIRS was recorded using 39 LED sources and 31 detectors from the NIRScout system developed by ‘NIRx Medical Technologies’ (LLC, USA), whose theoretical montage was shown in Alemi *et al*.^54^ Each source emitted near-infrared light at two wavelengths 760 and 850 nm. An ‘EasyCap’ (Easycap GmbH, Germany) was used to hold the sources and detectors, and their position was registered with three fiducials (nasion and left/right pre-auricular point) and later digitized using the ‘FieldTrip’ toolbox.^55^ There were 122 channels in total whose source–detector distance was on average 29.9 mm (±6.5 mm). No short channel was present in the montage (removal of systemic components was performed with Principal Component Analysis (PCA—see further). Before starting the recording, the experimenters checked the automatic gains for all channels and attempted to move the hair out of the way to optimize skin-to-optode contact. These gains were not changed once the recording had started.

Note that for both EEG and fNIRS, the presence of the coil certainly created a small bump on the scalp, so the cap could lose contact with the scalp in the area closely surrounding the coil, a phenomenon absent in the NH group. This did affect data quality for fNIRS but EEG to a smaller degree (see further details on data analysis).

### Protocol

**Figure 1:**
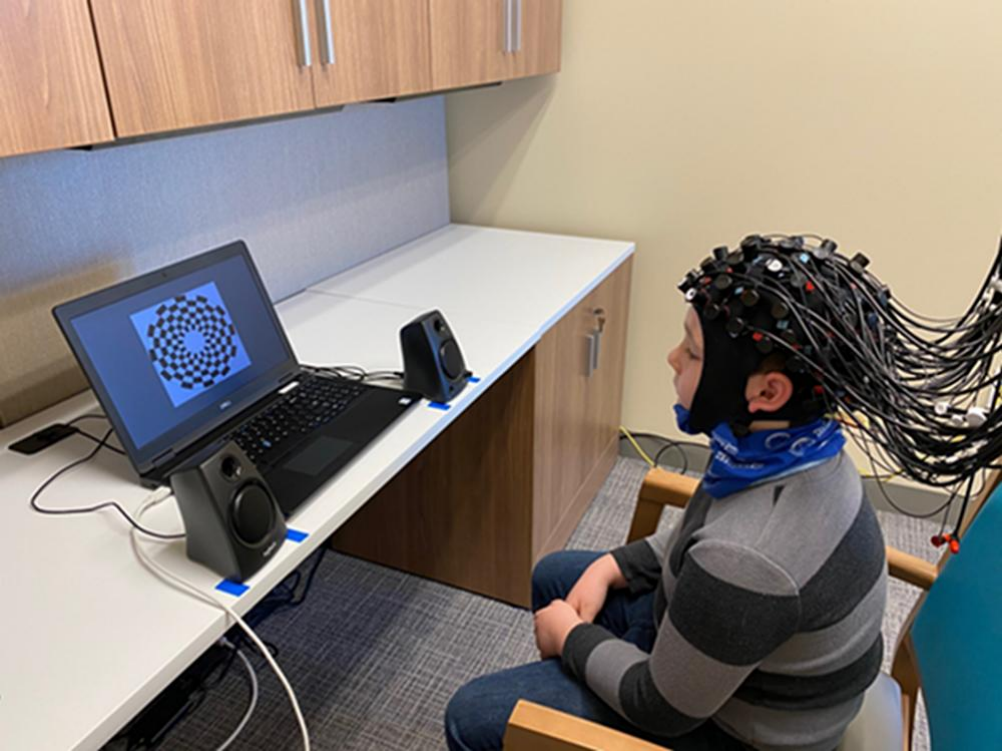
**Experimental protocol.** Experimental protocol depicted here with the fNIRS technique (the EEG setup being similar) as a child who wore CIs passively watched a monitor that displayed a rotating chequerboard in a 500-ms event design (for EEG) or a 15-s block design (for fNIRS). No sound was presented in this study, and implants were turned off.

The rationale for this research was explained to each child and their respective parents, and the entire protocol was described, after which parents and children provided informed consent. A battery of audiological tests along with language assessments were collected in addition to information pertaining to the implant and the progression of hearing loss. Children were then invited to sit still in a chair placed 1 m in front of a laptop on which the chequerboards were displayed (Fig. 1). The experimenters (co-authors J.W., S.N., J.M., L.H. and W.T.) placed proper caps after measuring the child’s head size. The data from the two imaging techniques were acquired sequentially, with counterbalanced order. Several other tasks were conducted using the same techniques in each child on the same day: a low-level auditory task,^56^ a motor task,^54^ a phonological task (spoken/written words and pseudo-words), an audiovisual integration task,^57^ emotional processing (a 10-min child-friendly video from the movie ‘Despicable Me’) and a 7-min resting-state recording.^58^ As the entire protocol for each technique was substantial, they were conducted at different times with a large break in between. Each participant was compensated financially for their participation, and the experiment was approved by the Western Institutional Review Board (reference #20190882).

### EEG data analysis and statistics

The recorded brain activity was analysed offline using ‘EEGLAB’^59^ and its ‘erplab’ plugin running under the ‘MATLAB’ (MathWorks Inc., MA, USA) environment. The 7-min resting-state recording and the 5-min chequerboard task were concatenated, the former being present only to help the Artifact Subspace Reconstruction method^60,61^ spot bad data periods. The events were adjusted by 50 ms to compensate for the delay between triggers sent to the ‘NetStation’ software relative to the occurrence of visual events. The data were re-referenced to the average of all 128 channels, band-pass filtered between 0.1 and 30 Hz with a second-order Butterworth filter and resampled at 256 Hz. Next, the ‘clean_rawdata’ plugin that implemented the Artifact Subspace Reconstruction method was used to correct bad data periods, and additional bad data periods were removed if they exceeded 7 SD (with at most 25% out-of-bound channels). There remained on average 163.9 ± 29.6 trials with a minimum of 83 and a maximum of 200 trials. This represented 82% of the initial data, and the statistical analyses on the number of trials excluded did not show significant difference between groups [*F*(2,72) = 3.1, *P* = 0.053]. A similar analysis was conducted for channel rejection: there were on average 119.1 ± 4.6 remaining channels with a minimum of 105 and a maximum of 128, representing 93% of the initial 128 channels, also with no group difference [*F*(2,72) = 0.4, *P* = 0.645].

The data were epoched from −200 to +1300 ms relative to the onset of the chequerboard, correcting for the baseline (−200 to 0 ms), and passed through an independent component analysis using the extended option of the ‘runica’ command and fed to the ‘ADJUST’ plugin.^62,63^ This algorithm did not favour nor penalize any particular group [*F*(2,72) = 2.6, *P* = 0.078]. There were on average 15.5 ± 6.6 independent components removed: 1.3 ± 2.8 were eye blinks, 4.5 ± 3.1 horizontal eye movements, 4.0 ± 3.1 vertical eye movements and 8.9 ± 5.2 generic discontinuities. Finally, all missing channels were spherically interpolated, and all epochs were averaged for each subject and each channel. The analysis was focused on two regions of interest (ROIs) that were selected using a set of electrodes to isolate the activity of the visual cortex and auditory cortex. For the visual cortex, a group of five electrodes surrounding Oz, namely, E75, E70, E83, E74 and E82, were selected according to the “EGI” nomenclature. For the auditory cortex, the bilateral superior temporal cortices were selected from E41, E46, E40 and E45 on the left side and E102, E103, E109 and E108 on the right side. The occipital lobe response consisted of several peaks, each extracted within a ±50 ms window centred around 130, 225, 350, 475 and 600 ms. The article focussed on the first peak where group differences were most striking, but subsequent peaks were also analysed (Supplementary Appendix A). The negative deflection in the waveform recorded over the temporal lobes was less systematic: rather than peak extraction, we took a conservative approach and simply averaged the potential over the presentation of the chequerboard from 0 to 500 ms.

A linear mixed-effect analysis (MATLAB’s fitlme function^64^) was conducted on the dependent variable (e.g. amplitude or latency) with two fixed factors: group and chronological age. It was necessary to include age because the waveforms measured in older children tended to be reduced (see ‘Discussion’—‘The role of chronological age’). For the same reason, all models included random intercepts by head size and by sex (both of which are known to affect VEPs^65,66^). Each main effect and interaction was tested by likelihood ratio tests progressively adding fixed terms to the final formula: DV ∼ group * age + (1| head size) + (1|sex). Finally, linear regressions were systematically conducted for age at first implantation and CELF score. Furthermore, we wanted to determine that the ERP waveforms elicited by the chequerboard and recorded from scalp electrodes over auditory areas had indeed an origin in the STG/middle temporal gyrus (MTG), so we performed a source analysis.^67-69^ We used the first ERP peak for each subject, separately for occipital and temporal regions and separately for the three groups. ERP source analyses were conducted using the minimum norm estimation for time-locked ERPs.^70,71^ The forward problem was solved using the volume conduction model using template MRI^72-74^ and the inverse solution with the minimum norm.^75^

### fNIRS data analysis and statistics

The fNIRS data were analysed using the ‘Brain AnalyzIR toolbox’.^76^ Step 1: the entire recording was trimmed 5 s before the first trigger and 5 s after the last trigger so that the selection of good/bad channels was based exclusively on the signal quality during the task. Step 2: oversaturated channels were replaced with high variance noise. Step 3: bad channels were flagged if their standard deviation over the trimmed signals (averaged over the two wavelengths) exceeded 15%. There were on average 18.4, 15.9 and 6.7 bad channels (out of 122) in groups LL, HL and NH, respectively, and this main effect of group was significant [*F*(2,72) = 6.6, *P* = 0.002] driven by fewer rejections in the NH group compared to LL and HL groups (*P* = 0.003 and *P* = 0.021, respectively), while LL and HL did not differ (*P* = 0.736). This means that NH children exhibited cleaner signals than children with CI, and we conjectured that this was related to the presence of the magnetic coil reducing the scalp-to-optode contact in some areas. All flagged channels were linearly interpolated from adjacent good channels. Step 4: signals were converted to optical density.^77^ Step 5: motion artefacts were corrected using Temporal Derivative Distribution Repair on the data that was first projected onto a PCA space before projecting back to the optical density space.^78^ Step 6: optical density signals were converted into changes in oxyhaemoglobin (HbO) and deoxyhaemoglobin (HbR) concentration using the modified Beer–Lambert law (based on extinction coefficients^79^) and based on source–detector distances calculated from the digitized montage specific to each child. The differential path–length factors were set at 7.25 and 6.38 for the 760 and 850 nm wavelength, respectively, and the absorption coefficients (µa, mm^−1^ M^−1^) were the following: µa (HbO, 760 nm) = 134.9, µa (HbO, 850 nm) = 243.6; µa (HbR, 760 nm) = 356.6 and µa (HbR, 850 nm) = 159.1, implemented in the toolbox. Step 7: Hb signals were band-pass filtered between 0.01 and 0.25 Hz to limit the low-frequency drift and cardiac oscillations. Step 8: Hb signals were passed through a PCA, and the first component was systematically removed. Screening through the responses of each child successively, we found it to be a more efficient way to remove the systemic component of the signals than a spatial filtering method.^80^

The subject-level statistics was performed by the ‘AR_IRLS’ function of the toolbox, using the default properties of the canonical haemodynamic response function.^81,82^ Group-level statistics followed a mixed-effect approach defined as follows: beta ∼ −1 + cond:Group + (1|Subject). The statistical maps in 3D were projected on the average digitized montage, and ROIs were isolated. The Talairach atlas was used to label brain regions for each channel, based on the probability that a given channel overlapped with a known cortical region (along the same reasoning as the fOLD toolbox^83^). A total of 20 channels overlapped with the primary visual cortex (V1) and visual association cortex (V2) in different proportions between 28.4 and 99.4% resulting in a weighted average for the visual ROI. The second ROI was selected to overlay with the auditory cortex. A total of 18 channels were partially overlapping with the STG (Brodmann area 22) with proportions varying from 20.7 to 32.1%, and 26 channels were overlapping with the MTG (Brodmann area 21) with proportions varying from 21.6 to 69.1%. Group averages of HbO and HbR waveforms were calculated after baseline correction (using 5 s prior to the chequerboard onset) for visualization purposes, but the generalized linear model analysis was entirely conducted on the weighted beta values mentioned above. ANOVA with one between-subject factor (groups LL, HL and NH) was conducted in each ROI on the beta values for HbO and HbR. Using the difference in HbO–HbR (to limit the inflation of Type 1 error), regression analyses systematically investigated the effect of chronological age, age at implantation and CELF score.

## Results

### EEG findings

**Figure 2:**
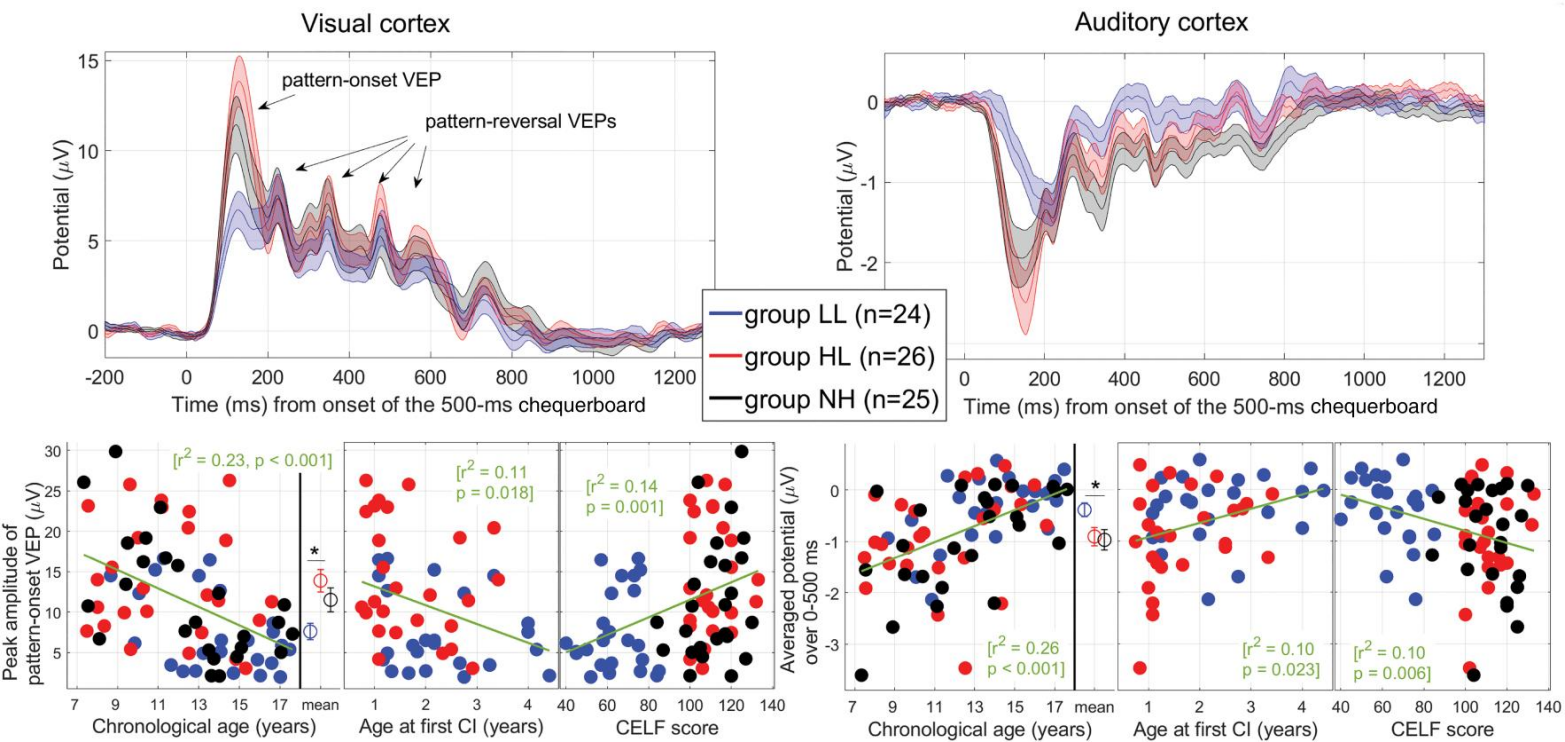
**EEG data measured over occipital and temporal electrodes.** Visually evoked potential recorded over occipital (*top*-left) and temporal (*top*-right) electrodes, averaged across repetitions of a flickering chequerboard presented every 1500 ms. Amplitude of the activation in each region plotted as a function of the child’s chronological age, their age at implantation and their language outcomes (*bottom*). Asterisks denote significant group differences obtained from the linear mixed-effect analysis. Lines are the fits from simple linear regressions, with their respective *r*2 and *P*-values.

The repetitive presentation of the chequerboard elicited a large response in the occipital lobe and a weak response (with opposite polarity, when referenced to the average) over the superior temporal cortices (Fig. 2). The group-averaged occipital waveforms had a large initial peak occurring around 120–130 ms, representing a ‘pattern-onset VEP’ from the grey background that preceded every visual event, followed by more modest peaks occurring roughly at 225, 350, 475 and 600 ms. The 125-ms periodicity was not a coincidence; it matched the rotation of the chequerboard, suggesting that these peaks were ‘pattern-reversal VEPs’, occurring roughly 100 ms after each reversal.

### Visual cortex

The first peak of the occipital response (i.e. pattern-onset VEP) was the most striking feature differentiating the groups. We also examined the topographical maps for the later peaks (i.e. pattern-reversal VEPs), but these analyses failed to find group differences (Supplementary Appendix A). So, we focus here on the first peak. The linear mixed-effect analysis revealed a main effect of group on P1 amplitude [*χ*^2^(2) = 11.7, *P* = 0.003], driven by smaller peaks for the LL than the HL group (*P* = 0.009). Peaks were on average 7.6, 13.9 and 11.5 µV, respectively, in LL, HL and NH groups (bottom-left, Fig. 2). There was also a main effect of chronological age [*χ*^2^(1) = 15.4, *P* < 0.001], with a linear trend showing a reduction of 11.4 µV in a decade (explaining 23% of the variance). However, there was no interaction between age and group [*χ*^2^(2) = 4.3, *P* = 0.117]. For P1 latency (not shown) that averaged at 123.9 ms, there was neither a main effect of group [*χ*^2^(2) = 4.4, *P* = 0.110] nor a main effect of chronological age [*χ*^2^(1) = 0.3, *P* = 0.620], without interaction [*χ*^2^(2) = 2.2, *P* = 0.338]. Among children with CI, we found that both amplitude and latency of the pattern-onset VEP decreased with age at implantation (*r*^2^ = 0.11, *P* = 0.018 and *r*^2^ = 0.08, *P* = 0.044). Finally, amplitude (but not latency) positively correlated with the CELF score (*r*^2^ = 0.14, *P* < 0.001) in line with group differences.

### Auditory association areas

**Figure 3:**
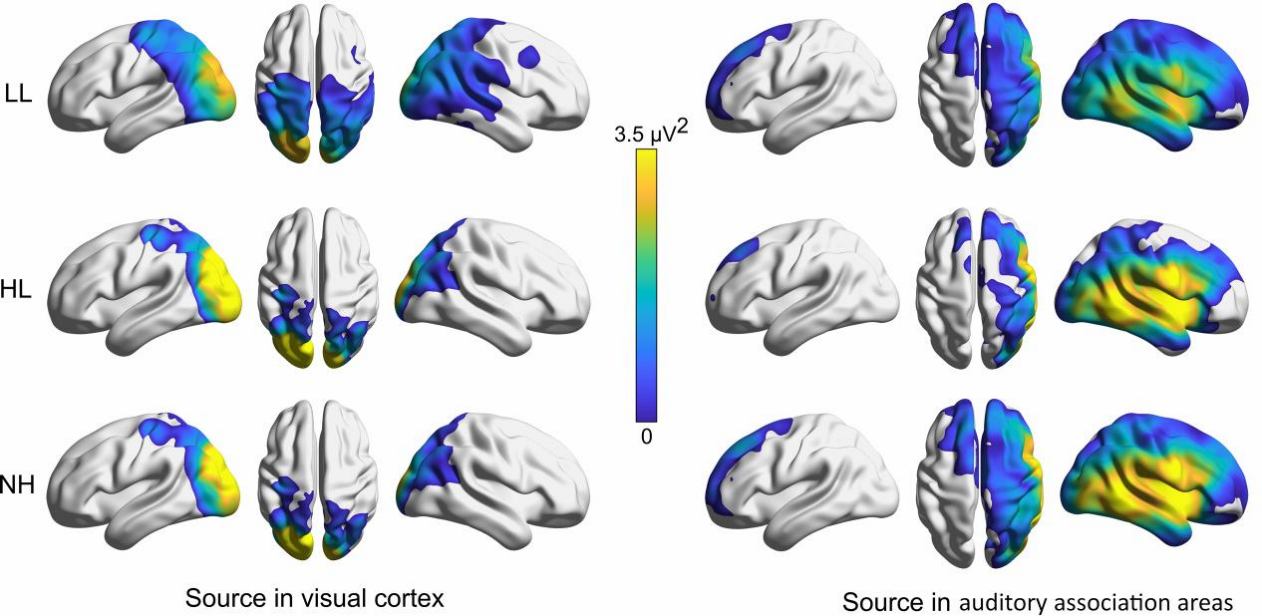
**Source localization results from EEG data.** Power at the sources obtained at the pattern-onset VEP for each group of children (LL on *top*, HL in the *middle* and normal hearing on the *bottom*). Critically, the response captured at the scalp over MTG/STG electrodes had an auditory origin (and not a by-product of the visual cortex response).

Negative potentials were observed over the temporal region electrodes during the chequerboard presentation. The linear mixed-effect analysis revealed a main effect of the group [*χ*^2^(2) = 6.2, *P* = 0.046], driven by stronger activity (i.e. more negative potentials) for the HL and NH groups compared to the LL group (*P* < 0.040). The average potential amplitude was −0.38, −0.90 and −0.97 µV, respectively, in LL, HL and NH groups (bottom-right, Fig. 2). There was also a main effect of chronological age [*χ*^2^(1) = 19.4, *P* < 0.001], without interaction [*χ*^2^(2) = 1.5, *P* = 0.483]. The potentials were weaker (i.e. less negative) in older children, with an estimated slope of 1.6 µV per decade (explaining 26% of the variance). Among children with CIs, this synchronized activity was progressively lost with later implantation (*r*^2^ = 0.10, *P* = 0.023) and stronger for the children with better language outcomes (*r*^2^ = 0.10, *P* = 0.006; most-right bottom, Fig. 2). Importantly, the source localization analysis over the STG/MTG (Fig. 3, right panel) demonstrated that the waveforms recorded over temporal electrodes were not simply a by-product of the visual cortex response but had genuinely a cross-modal origin.

### Summary of EEG findings

The chequerboard task elicited a strong activity in the visual cortex, synchronized with a modest activity over auditory association areas. The occipital response was arguably complex, with each rotation of the chequerboard eliciting its own visual event resulting in additional peaks in the waveform spaced every 125 ms. Yet, this complexity could be broken down by isolating the first peak as a pattern-onset VEP that was dependent on the group, while later peaks were several instantiations of pattern-reversal VEPs which were not greatly affected by the group (Supplementary Appendix A). The weaker visual cortex response exhibited by the LL group provides support ‘against the hypothesis of compensatory changes’. In middle/superior temporal cortices, potentials were negative at the scalp (with average referencing) and had an origin separate from the visual cortex, suggesting that this region was engaged in tandem but perhaps in opposite direction (see further discussion on whether this activity is truly inhibitory in nature) to the visual cortex. Critically, this coupling (or inverse coupling) appeared broken to some degree in the LL group, suggesting difficulties to engage a multimodal network in this task.

### fNIRS findings

**Figure 4:**
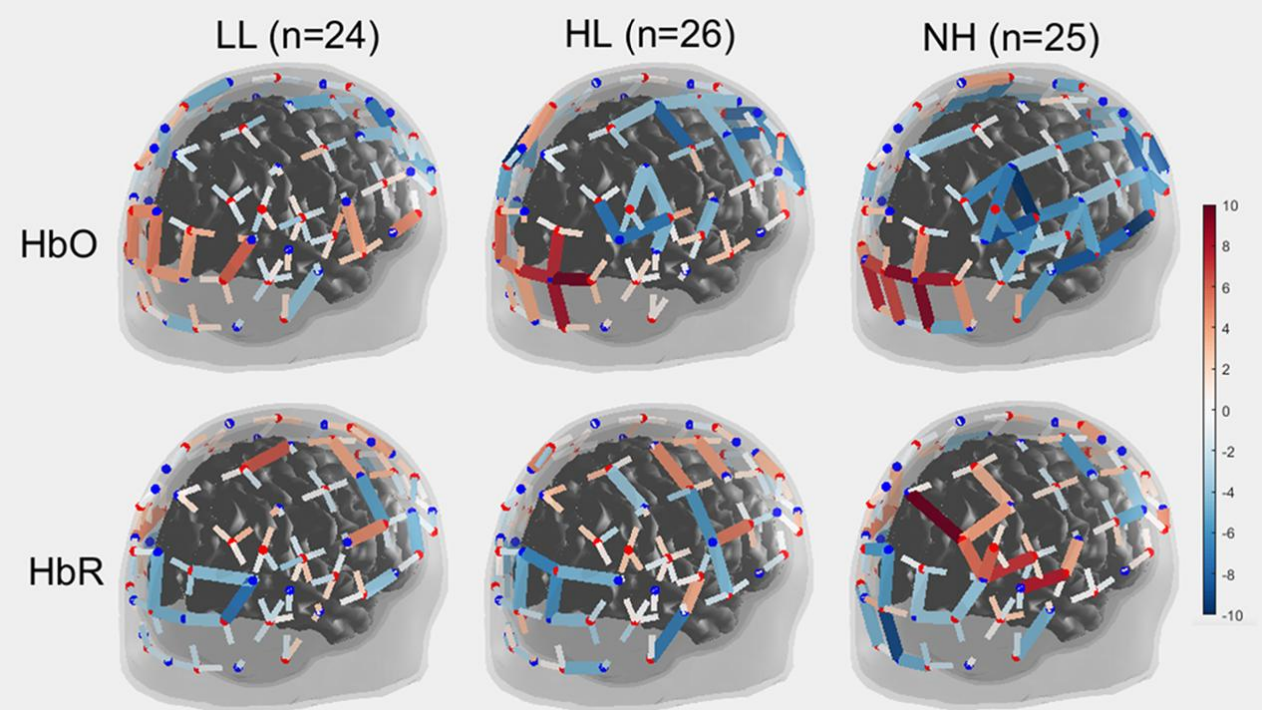
**fNIRS data for each channel of the montage.** 3D map of *t*-statistics on the beta weights obtained for the effect of the chequerboard versus rest, in each group, for oxygenated haemoglobin (*top*) and deoxygenated haemoglobin (*bottom*).

The chequerboard elicited a strong occipital response, revealed by a significant increase in HbO and a significant decrease in HbR in occipital channels (Fig. 4). Apart from visual areas, the rest of the brain either showed little change or was deactivated. Frontal and parietal regions were uncorrelated with the occipital activity, but both motor and temporal regions were anti-correlated with the occipital activity. The deactivation of the superior and middle temporal cortices was especially evident in NH children, much less so in CI children.

### Visual cortex

The ANOVA did not support a main effect of the group [*F*(2,72) = 0.6, *P* = 0.556 for HbO; *F*(2,72) = 1.6, *P* = 0.216 for HbR]. Individual values of HbO–HbR did not depend on chronological age (*P* = 0.339) and did not relate to language outcomes (*P* = 0.253). For children with CIs, these values did not relate to age at implantation (*P* = 0.392).

### Auditory association areas

**Figure 5:**
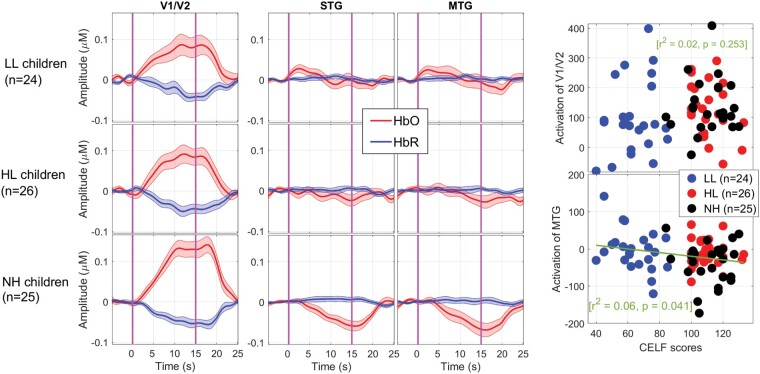
**Haemoglobin waveforms measured over occipital and temporal regions.** Group-averaged event-related changes in oxygenated and deoxygenated haemoglobin occurring in the visual cortex (left) or STG and MTG (*middle*). Vertical lines illustrate the onset (0 s) and offset (15 s) of the chequerboard presentation. Individual beta weights (HbO–HbR) over the visual cortex did not relate to language outcomes (*top*-right) but those recorded over the auditory association areas did to some extent (*bottom*-right). Lines are the fits from simple linear regressions, with their respective *r*2 and *P*-values.

There was a main effect of group over STG driven by changes in HbO [*F*(2,72) = 5.1, *P* = 0.009] but not in HbR [*F*(2,72) = 0.7, *P* = 0.507]. Similarly, there was a main effect of the group over MTG driven by changes in HbO [*F*(2,72) = 3.8, *P* = 0.028] but not in HbR [*F*(2,72) = 0.3, *P* = 0.769]. *Post hoc* comparisons clarified that the group effect (on HbO) was driven by a stronger deactivation in the NH group compared to both LL and HL groups (*P* < 0.030), which did not differ from each other. Chronological age did have a role over STG [*r*^2^ = 0.11, *P* = 0.003; but not over MTG (*r*^2^ = 0.03, *P* = 0.144)] suggesting that younger children were more prone to deactivate auditory regions than older children. This deactivation did not relate to age at implantation (*P* = 0.664 and *P* = 0.889, respectively, in STG and MTG) but tended to be associated with better CELF score (*P* = 0.086 over STG and *P* = 0.041 over MTG; bottom-right, Fig. 5).

### Summary of fNIRS findings

As expected, the visual task generated strong occipital activity revealed both by HbO and HbR signals. Unfortunately, group differences were not significant over V1/V2 (but are perhaps better appreciated in terms of spread—Supplementary Appendix B). The same applied to V3 or the fusiform gyrus (not shown) where the response was reduced in all groups. So, the visual ROI in this task was not helpful in addressing our competing hypotheses, but the auditory ROI was. NH children consistently deactivated the STG/MTG, while this trait was largely absent in children with CI and yet would have been desirable given its association to language outcomes.

## Discussion

The goal of this study was to explore phenomena of cross-modal plasticity by taking advantage of a population whose brain had gone through a first round of plastic changes under auditory deprivation and a later round of plastic changes once their hearing had recovered through electrical stimulation of their auditory nerve. As some aspects of visual and auditory networks rely on sensitive periods for spoken language and that some of them would be missed due to auditory deprivation, then one might expect both cortical regions to take on a differential developmental trajectory than for NH children. Furthermore, this could be mitigated by inherent language aptitudes^84^ if language was a key driver of the interactive specialization of visual and auditory networks.

More specifically, we suspected that children with CI would exhibit signs of cross-modal recruitment of auditory association areas by vision, i.e. left-over marks from the first round of plastic changes which had not completely reverted since CI experience. The fNIRS data did provide support for it as children with CI ‘failed to inhibit auditory association areas’, while this inhibition was a clear trait exhibited by NH controls. The EEG data converged to a similar idea but pointed to a reduced communication between visual and auditory cortices (i.e. a more unimodal response) and particularly targeted the children with poor language. Furthermore, our data generally support the idea that these plastic changes are deleterious or maladaptive on the basis that this inverse auditory–visual coupling relates to better language outcomes (bottom-right of Figs 2 and 5). So, lacking it (i.e. visual and auditory networks working independently) is not a good sign. Evidently, the brain cannot know what the world will be like tomorrow, e.g. suspecting that it will cease to be silent. So, networks may specialize for an environment that could become outdated.

As for the visual cortex, the present data call for caution when interpreting the pattern of responses and bearing in mind the technique with which observations were made. In EEG data, other than age effects (discussed below and were controlled for so that they did not impact group differences), a declining occipital response may be the result of adaptation.^52^ Whether this can be framed as a sign of ‘efficiency’ is doubtful. Visual adaptation would be revealed by a high first peak followed by rapidly decreasing peaks. We did observe the highest pattern-onset VEPs in HL children but negligible difference in subsequent peaks (Supplementary Appendix A). Even if we were to interpret this finding as children in the HL group having a more efficient visual system, then by the same reasoning, children in the LL group would have the least efficient visual system. Yet, the LL group had (on average) longer periods of auditory deprivation, so we must conclude that their brain did not promote vision. Similarly for the haemodynamic results, if the response magnitude reflected a form of mental energy consumption (which would be best kept as low as possible in a low-level task devoid of communication purpose), then both CI groups have not figured out an efficient way to save cognitive resources. Thus, the fNIRS data do not agree on an interpretation based on efficiency or resource allocation. Paradoxical as it may be, auditory deprivation did not result in a more efficient visual system, and this was especially true for children with poor language outcomes.

Similar paradoxes and inconsistencies have been raised in the literature and could partly be due to the profile of CI users. Weaker visual responses have been reported in post-lingually deafened CI adults,^33,34,52^ while stronger visual responses have been found in pre-lingually deafened CI children.^85^ But even within a population that shares much similarity, results are inconsistent. In the study by Campbell and Sharma,^85^ the 5–15-year-old children were comparable to the present LL group (given their age at implantation and speech scores), and they obtained earlier and larger VEPs compared to NH children, while we observed the contrary. Of course, there are methodological differences, as their visual stimulation could have engaged more connections to the rest of the brain, while ours was perhaps more prone to adaptation effects. This could explain why plastic changes take on distinct profiles across studies: targeting the right temporal cortex^85^ or posterior parietal regions but not the auditory cortex (in deaf adults without CI^86^) or a more complex network including prefrontal and parietal regions (in post-lingually deafened CI users^87^). We did not see clear evidence that parietal regions were recruited here, but the more unimodal activity observed here in some CI children (or lack of auditory–visual coupling) speaks to the importance of an integrated multimodal network even in response to a low-level visual task.

### Localized versus spread activity

Pattern-onset VEPs are generally known to be larger in amplitude than pattern-reversal VEPs, and their latency differs by about 125 versus 100 ms, respectively,^51,88,89^ although this depends somewhat on spatial and temporal frequencies.^90^ Here, we replicated this difference in amplitude (about 11 versus 6 µV) and average latencies of 124 versus 98 ms. The two visual stimulations are thought to activate different neural generators. Pattern-reversal VEPs are supposedly generated from V1 exclusively,^91^ whereas pattern-onset VEPs have multifocal generators, some of which at V2 or higher visual areas.^51,92,93^ The fact that we observed group differences in pattern onset but not in pattern reversal suggests that activity in V1 was similar across groups, but ‘weaker’ in the LL group in ‘more peripheral’ visual areas. This was corroborated to some degree by fNIRS data: the LL group’s response faded in amplitude relatively quickly from the single channel located over V1 to more peripheral channels of the occipital lobe, while it was more maintained spatially in HL and NH children (see Supplementary Appendix B). This narrowing of the cortical activation in a low-level task is a feature predicted by the interactive specialization framework^12^ and one that would deserve further exploration. But this differential spread of activity serves an important warning: whether one concludes of enhanced or impaired response to a given stimulation is evidently dependent on the size of the ROI chosen (in addition to the task dependency aforementioned).

### The role of chronological age

Although not directly related to our competing hypotheses (compensatory or deleterious cross-modal changes), a consistent observation made throughout our EEG data was the progressive reduction in amplitude measures for older children, in line with previous findings.^94^ The morphology of ‘pattern-reversal VEP’ is supposed to be adult-like in the first couple years of life.^95,96^ In contrast, the morphology of ‘pattern-onset VEP’ takes on a longer developmental course extending into adolescence and even adulthood.^89,97,98^ For this reason, effects of chronological age were expected for the pattern-onset VEPs (and controlled for in the analyses), but we observed them in pattern-reversal VEPs as well. Perhaps, one explanation is head growth. With the increase in the head size, skull density and thickness, the electrodes at the scalp naturally become further away from the neural generators. As a result, VEPs recorded on older children are more likely to be reduced.^99^ Note that these considerations extend to sex differences.^38,66^ In this study, male and female children did not differ by age (*P* = 0.641), and this did not interact with group (*P* = 0.092), but we still controlled for it with random intercepts. Factors such as myelination and dendritic branching complicate the issue by occurring at a different pace in different brain regions, often coinciding with functional development.^100^ As cortical areas become more fully myelinated, larger potentials might be facilitated in older children, thereby counteracting the effect of the head size. The plasticity of myelination (see, for example, Smith *et al*.^101^ for a lack of myelination in the context of auditory deprivation in infants) also might have impacted the amplitude and latency of different components of a VEP, differentially for young versus older children and perhaps differentially for NH versus CI children.

In fNIRS methodology, age effects are less systematic because different factors counteract each other. The ‘banana-shaped photon path’ of NIRS should theoretically go deeper on a smaller head.^102,103^ On this basis, it should be easier to measure oxygen demands caused by neural activity in younger children. On the other hand, the cerebral blood flow and the oxygen metabolism exhibit developmental changes^104^ such that oxygen demands elicited by a given task may not be as obvious in young children. Problematically, these developmental changes are once again not homogeneous across brain regions. Here, responses were reduced in older children over STG, but this did not happen in the visual cortex. Maybe the deactivation of auditory regions is genuinely facilitated in younger children, but this point should be taken with great caution until we understand the root cause of these age effects.

### Methodological choices and limitations

A number of methodological choices made in this study deserve further justification, along with acknowledgement of the techniques’ limitations.

#### Task

Chen *et al*.^52^ noticed that studies that engaged participants in an active task (e.g. visual discrimination) tended to reveal ‘stronger’ visual cortex response by deaf individuals or CI users relative to NH controls.^105-107^ In contrast, studies that presented visual stimuli repeatedly (eliciting visual adaptation) tended to report the opposite.^33,86,108^ Considering that 7-year-old children would generally not pay as much attention as 18-year-old children, and given the low-level nature of a flickering chequerboard, we thought it was simpler to take attention out of the equation in this cohort and use a passive task. This choice might have (potentially) facilitated a weaker response by CI children in the LL group, but CI children in the HL group exhibited ‘stronger’ visual cortex response, hinting at the idea that visual adaptation would interact with language skills. Also note that we replicated this contrast (and the weak visual cortex response of children in the LL group) with more advanced forms of visual stimulation (a speaking face in Alemi *et al*.^57^ and written words/sudowords in another article in preparation). So, it is very unlikely that the passive nature of the current task was the main reason for a weak visual cortex response in the children of the LL group.

#### Sample size

Many CI studies that included neuroimaging have ranged from 10 to 30 participants. Furthermore, studies often targeted adult CI users, sometimes with very different onset and duration of deafness or CI experience, whose heterogeneity induced inconsistencies in cortical activations. This prompted us to target a more homogeneous population with (i) a relatively larger sample, (ii) younger ages with limited auditory deprivation (maximum of 4 years) and (iii) controlled educational outcomes. In a previous study, we showed that standard clinical tests (speech perception measures) corroborated the children’s global ease or difficulty with language, and we confirmed that age at implantation was (on a group level) a relevant factor but it did not account for individual variability.^26^ Here, we found group differences and correlations with individual outcomes, so we strongly suspect some of this variability to originate from cross-modal reorganization. Recent explorations of auditory cortex response to an oddball paradigm^56^ and resting-state networks^58^ in the same sample of children also revealed striking group differences and links to language and literacy outcomes, reinforcing the notion that this neuroimaging approach to CI outcomes is promising and has clinical relevance.

#### Limitation of EEG

In the EEG data, there is potential for misinterpreting the activity recorded over the STG (right panel, Fig. 2). At the scalp, activity from the auditory cortex is generally best captured around the vertex (Cz or FCz) due to the orientation of the respective dipole.^109^ The presence of a dominant potential at Oz (here as much as 15 μV) could result in negative potentials at the vertex, simply because each electrode was referenced to the average of all 128 electrodes. The same argument is held for temporal electrodes, casting a doubt as to whether the STG waveforms were genuinely initiated from auditory regions or a pale inverted version of the visual cortex response. This is why it was critical to conduct a source localization analysis^110,111^ that demonstrated an independent STG/MTG origin in this visual task. Other studies^65,85^ also recorded cross-modal VEPs, and their source analysis corroborated the idea of STG/MTG generators. Furthermore, the recruitment has been consistently stronger on the right side.^32-34^ Here, we also found a strong asymmetry pointing to the right auditory cortex being coupled with the visual cortex (Fig. 3, right panels). But it is genuinely difficult to assert this neural activity as ‘inhibitory’ in nature. The negative responses obtained with fNIRS over MTG/STG are certainly helpful in this regard as they support the idea of ‘deactivation or disengagement’, but as far as EEG is concerned, a safer interpretation is that of desynchronization of auditory association areas with the visual cortex activation. Finally, we should reiterate once more that it is not the primary auditory cortex that tends to be recruited during visual tasks but rather ‘auditory association areas’ (as observed from other techniques such as PET, MEG and fMRI). These areas are closer to the scalp; they have more varied dipole orientations; and this is perhaps why they may be relatively well captured by temporal electrodes. Thus, we believe these cross-modal changes to auditory association areas to be genuine and a promising indicator of the interplay between visual and auditory functions in children with CI, extending to language outcomes. In this regard, we aim to further explore this population with audiovisual speech materials to get a more comprehensive picture of the quality of multi-sensory communication in ecological settings. Especially if these plastic changes affect parietal regions,^86^ enhancement in multi-sensory integration would be extremely valuable to study (see review by Strelnikov *et al*.^112^ in the PET literature). Unfortunately, these cortical regions are often the ones contaminated by CI artefacts in EEG recording. Here, it is worth reminding that all devices were switched off since there was no sound, and this successfully limited the prevalence of such artefacts. But for more complex materials and tasks, this issue remains a challenge, and it is highly variable from one device to another,^113-115^ posing serious complications for enterprises such as EEG source analysis.

#### Limitation of fNIRS

**Figure 6:**
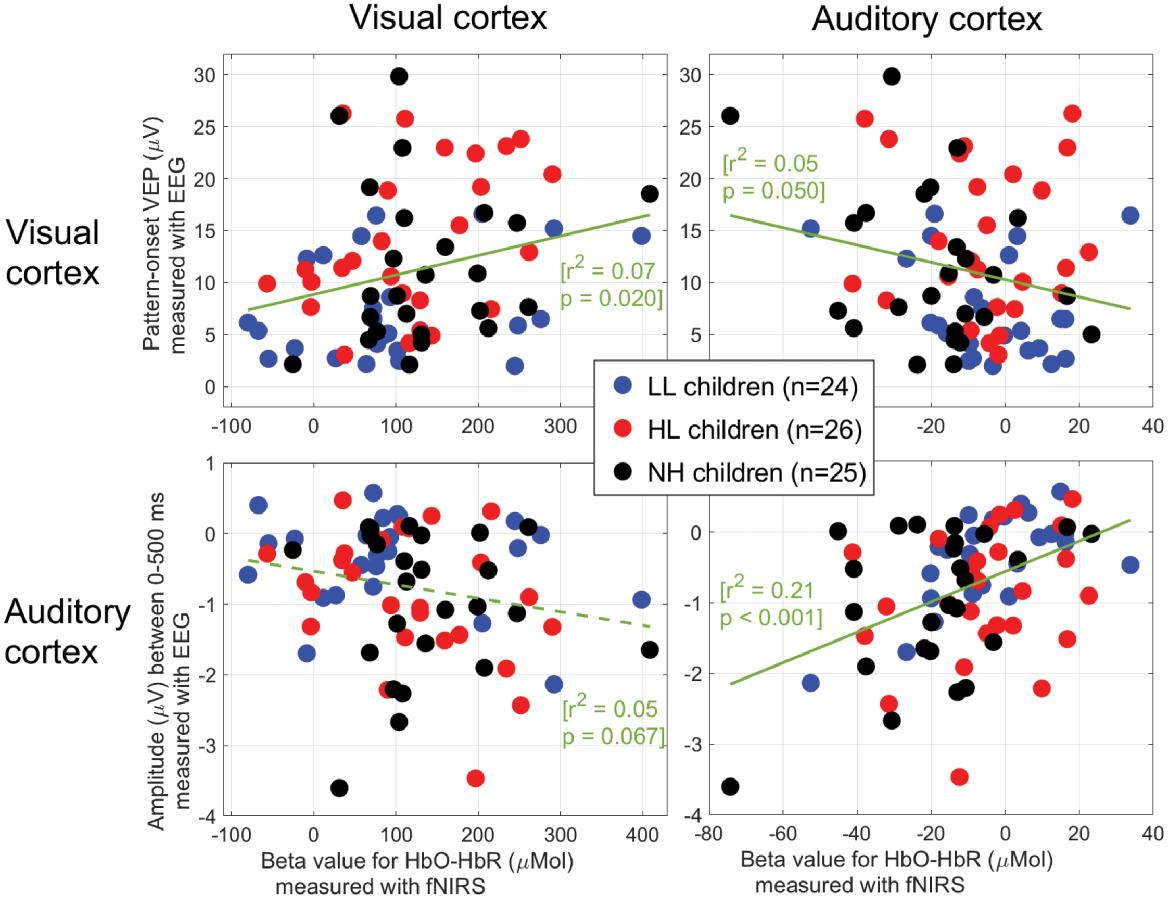
**Correlation of cortical activity across the two techniques.** Comparison of EEG and fNIRS metrics in visual and auditory cortices. Activation of the visual (*top*-left) and auditory (*bottom*-right) areas was consistent between the two techniques and inversely related to each other (*top*-right and *bottom*-left).

Even though fNIRS appears as a promising alternative, devoid of the CI artefacts aforementioned, this imaging technique has limitations of its own. First, it cannot capture activity from structures deep in the brain, so it might be hard, for example, to isolate activity from A1. This may not be an issue if the areas recruited are ‘auditory association areas’, closer to the scalp. But it certainly constrains the type of research questions that can be answered in CI users.^116,117^ The loss of optode contact to the scalp due to the coil is also problematic for studies specifically interested in the cortical area directly underneath. Furthermore, it is a pity that the technique simply fails in some subjects. Improving test/retest reliability (including for simple tasks such as a chequerboard^118^) and reducing the impact of skin pigmentation and hair type^119,120^ would be highly desirable for this technique to reach higher standards of neuroimaging. Currently, although our activation metrics were related between EEG and fNIRS (Fig. 6), the shared variance remained modest. Similar attempts at comparing fNIRS and EEG^121-123^ or fNIRS and fMRI (e.g. Arun *et al*.^124^) have also provided only modest agreement.

### Clinical significance

The idea that visual language reinforces cross-modal plasticity thereby compromising the functions of the auditory cortex has been questioned. In a review of this literature, Lyness *et al*.^125^ argued that there is in fact no evidence to link the use of visual language to poorer CI outcomes. Instead, they pointed towards the detrimental role of ‘language deprivation’ during sensitive periods. This debate has major implications because sign language is often discouraged in rehabilitation of children with CI. A similar conclusion was reached by Mushtaq *et al*.^43^ when observing through fNIRS that CI children displayed similar responses to auditory speech as NH children in the temporal cortex and even larger responses to visual speech than NH children. Thus, they recommended ‘encouraging the use of visual language’ in this paediatric population. The current findings do not allow us to comment on this debate with much certainty. Our current position is that the LL group exhibited a form of uncoupling between auditory and visual functions, which was generally detrimental to language. But further work is needed to better understand why recruitment of the auditory cortex during a visual task is (most often) ‘maladaptive’ as it was here, while recruitment of the visual cortex during an auditory task is (most often) ‘adaptive’, and the conditions in which this dichotomy may be found.^42,126,127^

## Conclusion

To this day, some children with CI struggle at school, despite early implantation and continuous rehabilitation efforts.^29,30^ Here, we provided converging evidence—using non-simultaneous EEG and fNIRS—that one reason for these ongoing difficulties is that the brain of these children has organized itself in a way that is not favourable to language development. These changes, surprisingly, did not seem to promote visual information, at least not in response to a simple chequerboard. One consistent marker observed across the two techniques was the desynchronization of superior and middle temporal cortices with the visual cortex. Not seeing this inverse coupling between visual and auditory functions may be an indication that the brain of some (but not all) children with CI is not tuned optimally to integrate linguistic stimuli that are intrinsically audiovisual.

## Supplementary Material

fcae175_Supplementary_Data

## Data Availability

The data and scripts supporting the findings of this article are available on this OSF repository: osf.io/zqynm/.
