# Supplementary material for: Cross-modal plasticity in children with cochlear implant: converging evidence from EEG and functional near-infrared spectroscopy
Source: Brain Commun. 2024 May 21;6(3):fcae175. doi: 10.1093/braincomms/fcae175 (PMC11154148; doi:10.1093/braincomms/fcae175)
Supplement: fcae175_Supplementary_Data [file fcae175_supplementary_data.pdf]

Supplementary Appendix A: Additional EEG analysis

We examined the topographical maps of each group at 225, 350, 475, and 600 ms, corresponding to each of the later peaks, i.e. several instantiations of pattern-reversal VEPs. Except for the peak at 225 ms, children in the HL group (middle column) exhibited a slightly stronger activity of their visual cortex than children in the LL group (left column).

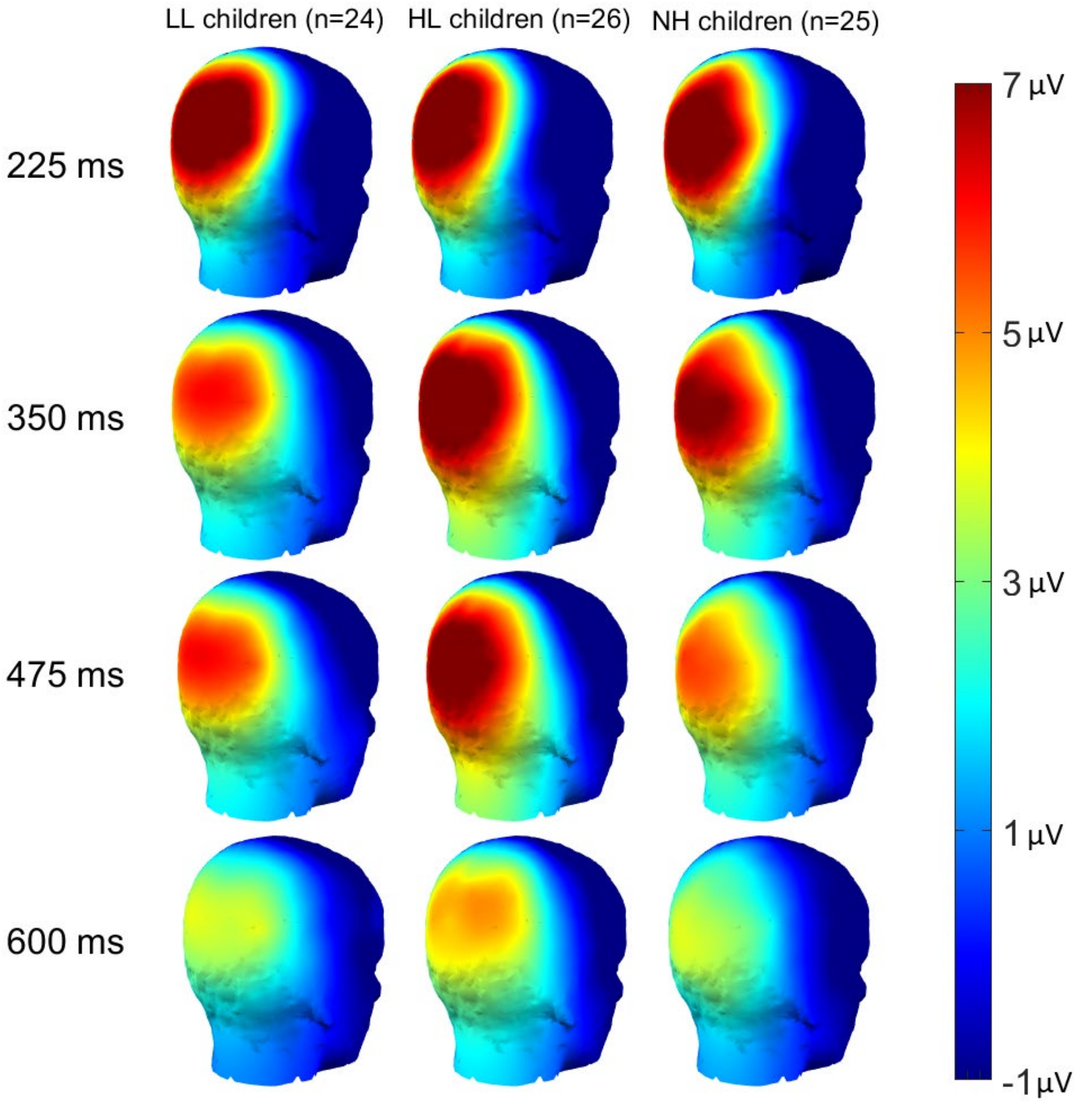

Supplementary Figure A1: Topographical maps of each group at times corresponding to subsequent occipital peaks in response to each reversal of the checkerboard.

Second peak (225 ms): For P2 amplitude, there was no main effect of group [$\chi^2(2)<0.1$, $p=0.982$], and the main effect of chronological age missed significance [$\chi^2(1)=3.0$, $p=0.083$], without interaction [$\chi^2(2)=2.3$, $p=0.310$]. The non-significant trend with age suggested a decrease in amplitude in older children. For P2 latency, there was no main effect of group [$\chi^2(2)=5.0$, $p=0.083$], no main effect of chronological age [$\chi^2(1)=1.5$, $p=0.221$], without interaction [$\chi^2(2)=1.7$, $p=0.428$]. Age at implantation did not matter for either amplitude or latency [$p>0.307$].

Third peak (350 ms): For P3 amplitude, there was no main effect of group [$\chi^2(2)=2.1$, $p=0.356$], but a main effect of chronological age [$\chi^2(1)=9.7$, $p=0.002$], without interaction [$\chi^2(2)=2.3$, $p=0.311$]. P3 amplitude decreased in older children. For P3 latency, there was no group effect [$\chi^2(2)=3.0$, $p=0.224$], no age effect [$\chi^2(1)=0.2$, $p=0.642$], and no interaction [$\chi^2(2)=2.2$, $p=0.332$]. P3 amplitude decreased (slightly) with age at implantation [$p=0.030$] but not P3 latency [$p=0.754$].

Fourth peak (475 ms): For P4 amplitude, there was no main effect of group [$\chi^2(2)=2.8$, $p=0.248$], but a main effect of chronological age [$\chi^2(1)=9.8$, $p=0.002$], without interaction [$\chi^2(2)=0.9$, $p=0.645$]. P4 amplitude decreased in older children. For P4 latency, there was no group effect [$\chi^2(2)=1.0$, $p=0.608$], no age effect [$\chi^2(1)=1.8$, $p=0.184$], and no interaction [$\chi^2(2)=0.4$, $p=0.824$]. Age at implantation did not matter for either amplitude or latency [$p>0.090$].

Fifth peak (600 ms): For P5 amplitude, there was no main effect of group [$\chi^2(2)=2.0$, $p=0.369$], and the main effect of chronological age missed significance [$\chi^2(1)=3.2$, $p=0.075$], without interaction [$\chi^2(2)=4.4$, $p=0.113$]. The non-significant trend suggested a decrease in P5 amplitude in older children. For P5 latency, surprisingly, there was a main effect of group

[$\chi^2(2)=6.9, p=0.032$], no main effect of chronological age [$\chi^2(1)=0.6, p=0.443$], without interaction [$\chi^2(2)=1.0, p=0.602$]. The group difference was driven by larger amplitude in the HL group compared to the LL or NH groups, but none of the pairwise comparisons actually reached significance, so we ignored it. P5 amplitude decreased (slightly) with age at implantation [$p=0.032$] but not P5 latency [$p=0.146$].

Averaged pattern-reversal VEP: Given the good consistency across these peaks, we can average their amplitude and express their latency relative to a given reversal of the checkerboard. The LME analysis confirmed the consistent pattern aforementioned, i.e. no main effect of group [$\chi^2(2) = 1.0, p = 0.601$], but a main effect of chronological age [$\chi^2(1) = 6.3, p = 0.012$] without interaction [$\chi^2(2) = 2.4, p = 0.303$]. The pattern-reversal VEPs were reduced by about 5.1 µV per decade, and the average peak was 6.3 µV within our age range (left, Figure A2). For latency, there was neither a main effect of group [$\chi^2(2) = 2.1, p = 0.341$], nor a main effect of chronological age [$\chi^2(1)<0.1, p = 0.753$], without interaction [$\chi^2(2) = 2.8, p = 0.242$]. On average, the latency of pattern-reversal VEPs was 97.6 ms (right, Figure A2). Neither amplitude nor latency reached a significant correlation with age at first implantation [$p = 0.097$ *and* $p = 0.166$, respectively]. The amplitude of the later peaks did not correlate with the CELF score [$p = 0.256$], but their latency did to some degree [$r^2 = 0.07, p = 0.018$]: children with shorter latencies tended to obtain better language outcomes. Nevertheless, given the lack of group differences on these pattern-reversal measures, this latter relationship was not emphasized in the article (which focused on the *pattern-onset VEP* instead).

Summary: The amplitude of each of these pattern-reversal peaks decreased in older children, presumably for physiological reasons such as skull growth and myelination (see Discussion "Role of chronological age"). Age, however, had no effect on their respective latency. Group differences were largely absent in either amplitude or latency. We conclude that, for some reason (perhaps because it reflects V1 activity more exclusively), the pattern-reversal VEPs do

not offer ample opportunity to differentiate between children with CIs, and thus would have limited audiological application, contrary to the pattern-onset VEP discussed in the main article.

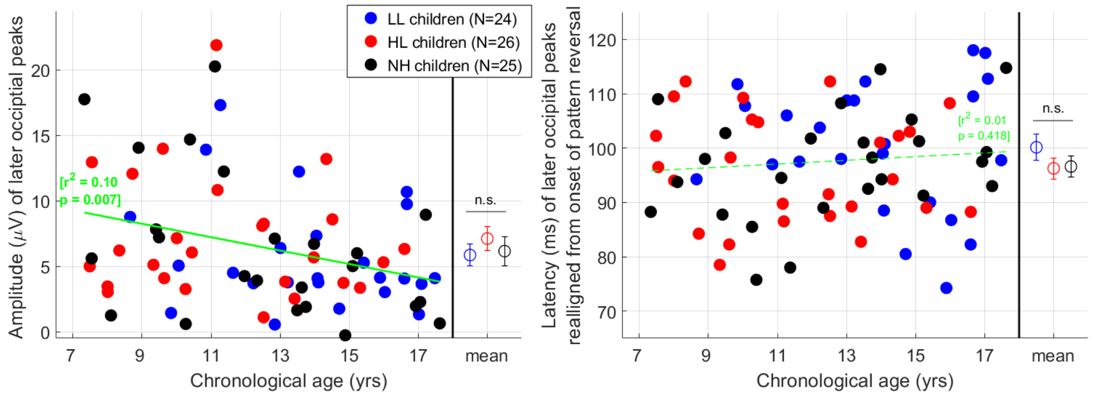

Supplementary Figure A2: Average amplitude (left) and latency (right) of peak 2 to 5 corresponding to a pattern-reversal VEP, plotted as a function of the child's chronological age. Note that the peaks occurred roughly 100 ms after a given rotation of the checkerboard (every 125 ms), so they were realigned here at each reversal onset, before averaging.

Supplementary Appendix B: Additional fNIRS analysis

Here, we delved more closely into several regions of the occipital lobe, from its centre (V1) to more peripheral areas. As illustrated in Figure B1 from left to right, we noticed that the group LL showed the most activation on V1 but it faded quickly in peripheral areas. In contrast, for children in the group HL and even more for NH children, the occipital response appeared to be maintained over peripheral regions.

One single channel at V1 (S22-D16): There was no main effect of group [$F(2,72)=2.6$, $p=0.083$ for HbO; $F(2,72)=0.7$, $p=0.506$ for HbR].

Six channels directly around V1: There was no main effect of group [$F(2,72)=0.1$, $p=0.895$ for HbO; $F(2,72)=0.3$, $p=0.762$ for HbR].

Three channels below Oz: There was no main effect of group [$F(2,72)=1.7$, $p=0.198$ for HbO; $F(2,72)=2.4$, $p=0.102$ for HbR].

Six channels towards inferotemporal regions

There was no main effect of group [$F(2,72)=1.9$, $p=0.151$ for HbO; $F(2,72)=2.0$, $p=0.146$ for HbR].

Four channels towards parietal regions: There was no main effect of group [$F(2,72)=0.2$, $p=0.803$ for HbO; $F(2,72)<0.1$, $p=0.976$ for HbR].

Summary: Although, there seems to be a differential spread of activity in the LL group compared to the HL and NH groups, there is too much heterogeneity to highlight group differences. This is why our analysis combined these 20 channels (completely non-redundant) which were all identified by the Tailarach atlas as partially overlapping with V1/V2.

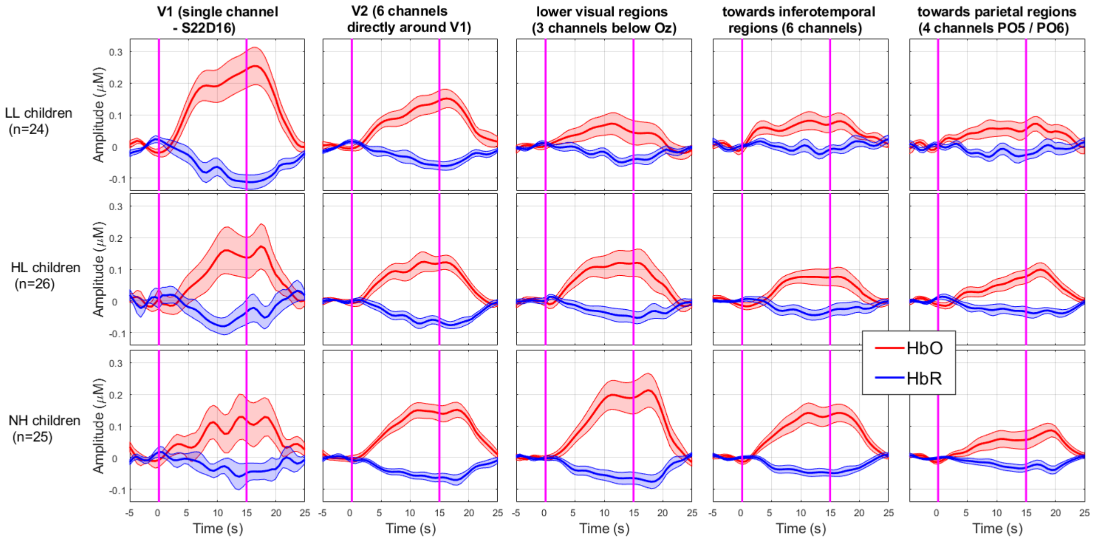

Supplementary Figure B1: Group-averaged event-related changes in oxygenated (red) and deoxygenated (blue) hemoglobin occurring in slightly different parts of the visual cortex, going from its centre (most-left) to more peripheral areas (towards right panels).
